# NF-**κ**B Inhibition after Cecal Ligation and Puncture Reduces Sepsis-Associated Lung Injury without Altering Bacterial Host Defense

**DOI:** 10.1155/2013/503213

**Published:** 2013-11-18

**Authors:** Hui Li, Wei Han, Vasilly Polosukhin, Fiona E. Yull, Brahm H. Segal, Can-Mao Xie, Timothy S. Blackwell

**Affiliations:** ^1^Division of Respiratory Medicine, The First Affiliated Hospital of Sun Yat-Sen University, Guangzhou, Guangdong Province 510080, China; ^2^Department of Cancer Biology, Vanderbilt University School of Medicine, Nashville, TN 37232, USA; ^3^Division of Respiratory Medicine, Nanjing Drum Tower Hospital & the Affiliated Hospital of Nanjing University, Nanjing, Jiangsu 210008, China; ^4^Department of Medicine, Division of Allergy, Pulmonary and Critical Care Medicine, Vanderbilt University School of Medicine, Nashville, TN 37232, USA; ^5^Departments of Medicine and Immunology, Roswell Park Cancer Institute, Buffalo, NY 14263, USA; ^6^Department of Medicine, School of Medicine and Biomedical Sciences, University of New York at Buffalo, Buffalo, NY 14214, USA; ^7^Department of Cell and Developmental Biology, Vanderbilt University School of Medicine, Nashville, TN 37232, USA; ^8^Department of Veterans Affairs Medical Center, Nashville, TN 37232, USA

## Abstract

*Introduction*. Since the NF-*κ*B pathway regulates both inflammation and host defense, it is uncertain whether interventions targeting NF-*κ*B would be beneficial in sepsis. Based on the kinetics of the innate immune response, we postulated that selective NF-*κ*B inhibition during a defined time period after the onset of sepsis would reduce acute lung injury without compromising bacterial host defense. *Methods*. Mice underwent cecal ligation and puncture (CLP). An NF-*κ*B inhibitor, BMS-345541 (50 *µ*g/g mice), was administered by peroral gavage beginning 2 hours after CLP and repeated at 6 hour intervals for 2 additional doses. *Results*. Mice treated with BMS-345541 after CLP showed reduced neutrophilic alveolitis and lower levels of KC in bronchoalveolar lavage fluid compared to mice treated with CLP+vehicle. In addition, mice treated with CLP+BMS had minimal histological evidence of lung injury and normal wet-dry ratios, indicating protection from acute lung injury. Treatment with the NF-*κ*B inhibitor did not affect the ability of cultured macrophages to phagocytose bacteria and did not alter bacterial colony counts in blood, lung tissue, or peritoneal fluid at 24 hours after CLP. While BMS-345541 treatment did not alter mortality after CLP, our results showed a trend towards improved survival. *Conclusion*. Transiently blocking NF-*κ*B activity after the onset of CLP-induced sepsis can effectively reduce acute lung injury in mice without compromising bacterial host defense or survival after CLP.

## 1. Introduction

Severe sepsis remains a challenging clinical problem that accounts for 1.3% of all hospitalizations and is the leading cause of mortality and morbidity in intensive care units [1, 2]. The most life-threatening complication of sepsis is the development of the multiorgan dysfunction syndrome (MODS), which most commonly affects the lungs and kidneys. Acute lung injury (ALI) occurs in approximately 30% of septic patients, and unfortunately there are no specific treatments available to reduce mortality other than low tidal volume ventilation [3]. 

Sepsis occurs when infections trigger the systemic inflammatory response syndrome in the host. As a central regulator of inflammation, nuclear factor-*κ*B (NF-*κ*B) has long been considered to be a critical pathway involved in the pathogenesis of sepsis [4, 5]. Available information suggests that inhibition of NF-*κ*B could be a promising therapeutic target in sepsis. A number of studies have investigated the effects of manipulating NF-*κ*B activity using genetic or pharmacologic approaches during sepsis and/or endotoxemia [6–12]. In general, NF-*κ*B downregulation has been successful in inhibition of inflammation and reduction of NF-*κ*B-mediated cytokines in these studies [6–9]. However, we and others have shown that NF-*κ*B plays an important role in host defense against bacterial infections in a variety of experimental models [13, 14], raising concern that therapeutic NF-*κ*B inhibition might have the unwanted effect of impairing bacterial clearance. A recent study showed that early neutrophil activation and infiltration (within 6 hours) are important for bacterial clearance and survival in septic animals [15]. Therefore, we reasoned that early NF-*κ*B activation and production of NF-*κ*B-dependent cytokines in sepsis might be sufficient for host defense, whereas prolonged NF-*κ*B activation could contribute to organ injury and dysfunction. To address this hypothesis, we investigated the impact of inhibition of NF-*κ*B activation for a defined period of time after the onset of sepsis. We used BMS-345541, a highly selective IKK inhibitor [16], for these studies. Due to its high oral bioavailability and relatively short half-life [16], BMS-345541 can be delivered orally and is effective for short-term inhibition of NF-*κ*B *in vivo*. 

## 2. Methods

### 2.1. Animal Model

Age- and gender-matched FVB mice between 8 and 16 weeks old were used for these studies. Mice were kept in a temperature-controlled room with light and dark cycles every 12 hours. All procedures were approved by the Institutional Animal Care and Use Committee at Vanderbilt University and complied with all state, federal, and NIH regulations. CLP was performed as previously described with minor modifications [17]. After mice were anesthetized with isoflurane (2-3%), the surgical area was disinfected with betadine and 70% alcohol and a midline laparotomy was performed. The cecum was then identified and the distal 50% of the cecum was ligated with 4-0 silk suture followed by puncture with one pass of a 21-gauge needle. The peritoneal cavity was closed using 5-0 nylon sutures. After surgery, 0.5 mL of sterile normal saline was administered by intraperitoneal injection. Sham laparotomy controls underwent the same surgical procedure without cecal ligation and puncture. Two hours after CLP, BMS-345541 (50 *μ*g/g body weight, obtained from Bristol-Myers Squibb) formulated in 3% Tween 80 and sterile water or an equivalent volume of vehicle (without BMS-345541) was administered by oral gavage [16]. Two additional doses of BMS-345541 or vehicle were then delivered at 6-hour intervals.

### 2.2. Survival Studies

Mice were subjected to laparotomy without CLP (Sham), laparotomy with CLP followed by vehicle delivered by gavage (CLP), and laparotomy with CLP plus BMS-345541 administration (CLP+BMS). Mice were evaluated daily and were euthanized at 7 days after CLP or earlier if they appeared moribund or in distress.

### 2.3. White Blood Cell Count and Differential

Peripheral white blood cell (WBC) counts were measured using a Coulter counter. Differential leukocyte counting was performed by manual counting of Wright-Giemsa-stained (Diff Quick; Baxter Scientific Products) peripheral blood smears.

### 2.4. Histology

After euthanasia, lungs were perfused with normal saline and inflated with 1 mL of 10% neutral buffered formalin. Lungs were then removed en bloc after tracheal ligation, preserved in 10% neutral buffered formalin for 24 hours at room temperature, and subsequently embedded in paraffin. Hematoxylin and eosin (H&E) stains were performed using standard protocol. The degree of parenchymal distortion in the alveolar tissue was assessed on 20 sequential high power fields per lung section and graded as follows: 0: normal; 1: <50% of interalveolar septa (IAS) have increased thickness due to edema and/or inflammatory cell infiltration; 2: >50% of IAS have increased thickness; 3: >50% of IAS have increased thickness and inflammatory cells are present within alveolar space; 4: consolidated infiltrate with distortion of normal alveolar architecture. The mean score was reported per section.

### 2.5. Bronchoalveolar Lavage Collection and Processing

Bronchoalveolar lavage (BAL) fluid was centrifuged at 400 g for 10 minutes to separate cells from supernatant. Supernatant was removed and stored at −70°C for cytokine determination. Cells were resuspended in cold PBS and total cell counts were measured on a grid hemocytometer. Cytocentrifuge slides were prepared and stained using a modified Wright stain. Differential cell counts were enumerated by counting at least 200 cells in sequential fields across the slide. 

### 2.6. Wet-to-Dry Ratio

The wet weight of lungs was determined immediately after excision. Lungs were then placed in an incubator at 65°C for 48 hours and the dry weight was recorded. 

### 2.7. Cytokine Assay and Nuclear NF-*κ*B Activity

Cytokines in BAL fluid and serum were measured using a Mouse Cytokine 20-Plex Panel (Invitrogen) that detects basic FGF, IL-1*β*, IL-10, IL-13, IL-6, IL-12, IL-17, MIP-1*α*, GM-CSF, MCP-1, IL-5, VEGF, IL-1*α*, IFN*γ*, TNF*α*, IL-2, IP-10, MIG, KC, and IL-4. Measurements of NF-*κ*B DNA-binding activity were performed on lung nuclear protein extracts obtained using NE-PER Nuclear and Cytoplasmic Extraction Reagents (Thermos). Ten *μ*g of nuclear protein extract from tissues was used for measuring DNA-binding activity of RelA (p65) (TransAM ELISA kit, Active Motif) according to the manufacturer's instructions.

### 2.8. Western Blots

Ten microgram of nuclear protein extract from lung tissue homogenates was separated on a 10% acrylamide gel (NuPAGE, Invitrogen). Western blot analyses were performed with antibodies against RelA (p65), TBP (Santa Cruz Biotech), and assessed using the Odyssey infrared system (LI-COR).

### 2.9. Bacterial Colony Counts

The right middle lobe, peritoneal fluid, and blood (obtained by cardiac puncture) were used for bacterial colony counts. Lung homogenates, peritoneal fluid, and serum were diluted into serial tenfold dilution using sterile PBS and plated on LB agar at 37°C for 24–48 hours, after which bacterial colonies were counted.

### 2.10. Phagocytosis Assay

RAW264.7 macrophages or cells obtained from peritoneal lavage at 3 hours after CLP were seeded into a 24-well plate at a concentration of 5 × 10^5^ cells/well. After attachment, cells were treated with *E. coli* lipopolysaccharide (LPS, 200 ng/mL), BMS-345541 (1 *μ*M), or LPS+BMS-345541. One hour after LPS treatment, *E. coli* DH5a, carrying the green fluorescent protein (GFP)-mut2 encoding plasmid pCD353 (*E. coli*-GFP), was added at a bacteria/leukocyte ratio of 20 : 1 and incubated at 37°C for 1 hour. Extracellular fluorescence was quenched by trypan blue, and the numbers of positive cells were counted using fluorescence microscopy, as previously described [18].

### 2.11. Statistical Analysis

To evaluate differences between groups, analyses were performed with GraphPad Instat software using an unpaired *t*-test (two groups) or ANOVA (multiple groups) followed by the Tukey-Kramer Multiple Comparisons posttest. Nonparametric ANOVA with Kruskal-Wallis posttest was used to evaluate histological scoring of lung injury. Survival data were plotted using Kaplan-Meier curves and analyzed by the log-rank test. Results are represented as mean ± SEM. Two-tailed *P* values <0.05 were considered significant.

## 3. Results

### 3.1. BMS-345541 Inhibits NF-*κ*B Activation after CLP

**Figure 1:**
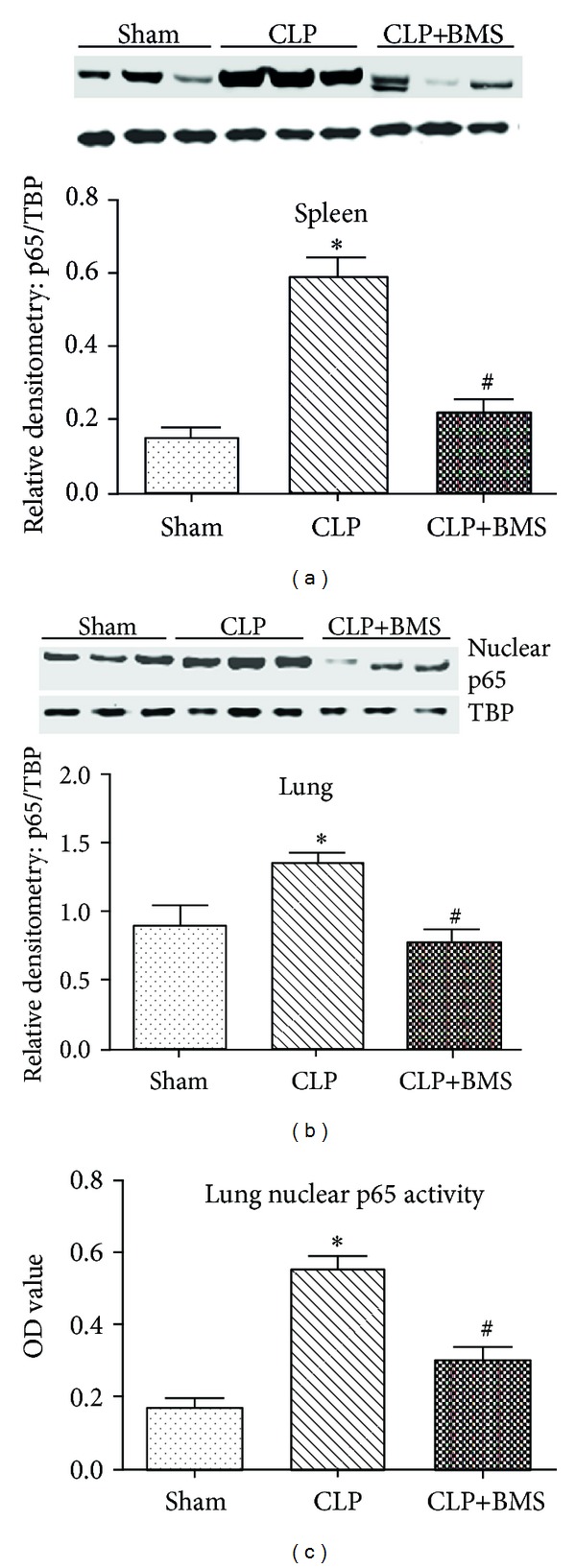
Treatment with the IKK inhibitor (BMS-345541) inhibits NF-*κ*B activation after CLP. Nuclear protein extracts from (a) spleen and (b) lung tissue were probed for p65 (RelA) by western blot. Densitometry shows p65 band density normalized by TATA binding protein (TBP). ANOVA test was used for comparison among multiple groups. Tukey post hoc tests were undertaken after ANOVA. There were significant differences for CLP versus sham or CLP+BMS mice (*P < 0.05 for CLP versus Ctrl, ^#^ *P* < 0.05 for CLP+BMS versus CLP), while there was no significant difference between sham and CLP+BMS mice. (c) Lung NF-*κ*B DNA binding activity was also assessed in nuclear protein extracts by TransAM ELISA. ANOVA test was used for comparison among multiple groups. Tukey post hoc tests were undertaken after ANOVA. There were significant differences for CLP versus sham or CLP+BMS mice (*P < 0.05 for CLP versus Ctrl, ^#^ *P* < 0.05 for CLP+BMS versus CLP), while there was no significant difference between sham and CLP+BMS mice. Results are presented as mean ± SE, *N* = 3 per group. Each experiment was repeated three times.

Based on previous work using BMS-345541 to block NF-*κ*B activation* in vivo* [6], we developed a protocol to target NF-*κ*B activation by beginning BMS-345541 treatment after the initiation of systemic inflammation following CLP. Mice were treated with BMS-345541 or vehicle by peroral gavage beginning at 2 hours after CLP. BMS-345541 dosing was repeated at 6-hour intervals for a total of three doses. Multiple doses were used because of the short half-life of the compound *in vivo* [16]. To evaluate the efficacy of this intervention, we measured nuclear RelA (p65) levels by western blot analysis at 24 hours following CLP. As shown in Figures 1(a) and 1(b), BMS-345541 treatment blocked RelA (p65) nuclear translocation in spleens and lungs at this time point. In addition, NF-*κ*B DNA-binding activity was reduced in the lungs of BMS-345541-treated mice compared to mice that received CLP and vehicle (Figure 1(c)). Together, these findings confirmed that BMS-345541 blocks CLP-induced NF-*κ*B activity in our model. 

### 3.2. BMS-345541 Limits CLP-Induced Neutrophilic Lung Inflammation and Prevents Lung Injury

**Figure 2:**
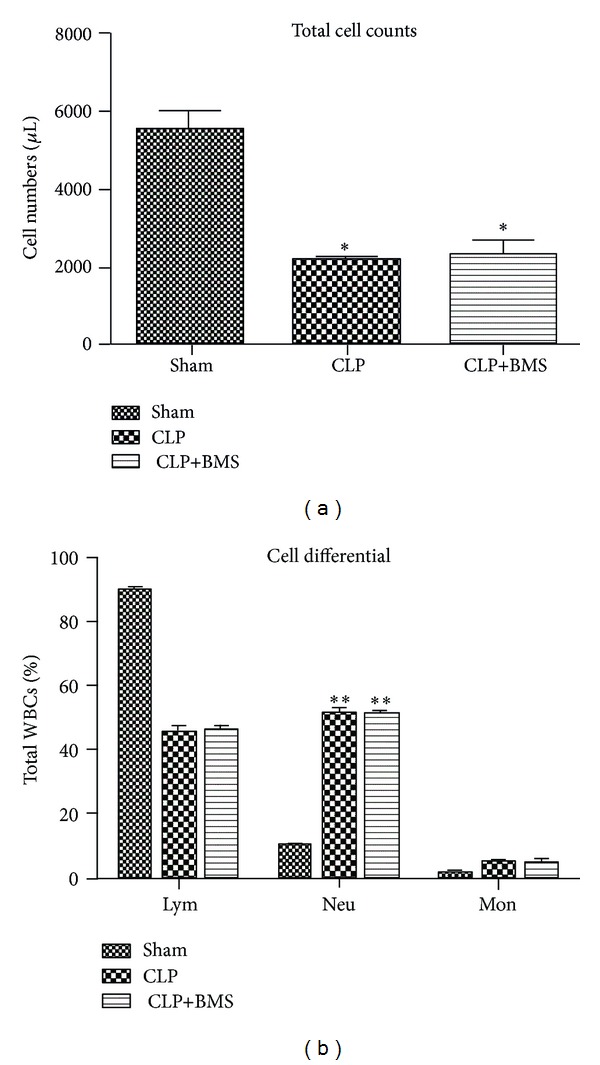
BMS-345541 treatment did not affect peripheral WBC counts in mice subjected to CLP. (a) Peripheral WBCs were counted in control mice treated with sham laparotomy (sham), mice that underwent CLP followed by vehicle (CLP), and mice treated with CLP followed by BMS-345541 (CLP+BMS) at 24 hours after surgery. *N* = 5 per group; ANOVA test was used for comparison among multiple groups. Tukey post hoc tests were undertaken after ANOVA. There were significant differences for sham versus CLP or CLP+BMS (*P < 0.05 for CLP and CLP+BMS versus sham), while there was no significant difference between CLP and CLP+BMS mice. (b) Differential WBC counts in mice with sham surgery, mice treated with CLP (+vehicle), and mice treated with CLP+BMS at 24 hours after CLP. *N* = 5 per group; ANOVA test (*P* < 0.0001) was used for comparison among multiple groups. Tukey post hoc tests were undertaken after ANOVA. There were significant differences for sham versus CLP or CLP+BMS mice (**P < 0.0001 for CLP and CLP+BMS versus sham), while there was no significant difference between CLP and CLP+BMS mice. Results are presented as mean ± SE.

After showing that BMS-345541 treatment attenuated NF-*κ*B activation, we investigated the impact of this treatment on CLP-induced lung and systemic inflammation. Consistent with prior studies [19, 20], CLP led to a decrease in peripheral WBC counts and an increase in the percentage of neutrophils at 24 hours compared to sham surgery controls (Figure 2). Treatment with BMS-345541 did not affect total or differential WBC counts at 24 hours after CLP. Similar findings were obtained from mice in the different treatment groups at 48 hours after CLP (not shown). 

**Figure 3:**
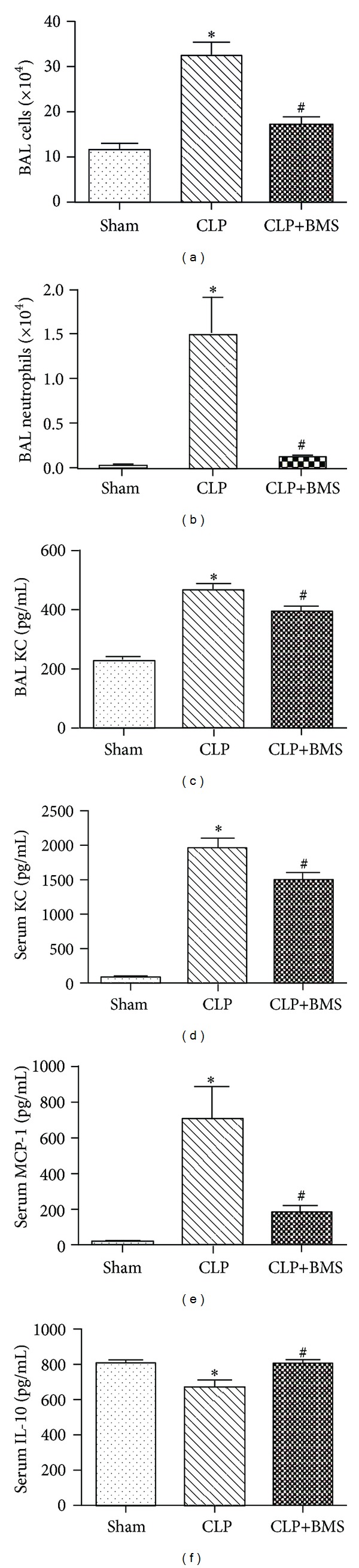
NF-*κ*B inhibition reduces lung inflammation after CLP. (a) Total cells, (b) neutrophils, and (c) KC levels in BAL at 24 hours after surgery in control mice treated with sham laparotomy (Ctrl), mice that underwent CLP followed by vehicle (CLP), and mice treated with CLP followed by BMS-345541 (CLP+BMS). *N* = 10 per group. *P < 0.05 for CLP versus sham laparotomy controls (Ctrl); ^#^ *P* < 0.05 for CLP+BMS versus CLP. (d) Serum cytokine levels for KC, MCP-1, and IL-10 at 24 hours after surgery. *N* = 5 per group. *P < 0.05 for CLP versus Ctrl; ^#^ *P* < 0.05 for CLP+BMS versus CLP. Results are presented as mean ± SE.

To evaluate the effects of NF-*κ*B inhibition on the course of lung inflammation in this model, we measured total inflammatory cells, neutrophils, cytokines, and chemokines in BAL fluid at 24 h after CLP. We found a significant reduction in the total number of BAL cells, as well as BAL neutrophils, in the CLP+BMS group compared to the CLP group (Figures 3(a) and 3(b)). Total BAL cells and neutrophils were similar between the CLP+BMS group and sham laparotomy controls. At 48 hours after CLP, we found a similar pattern of cells in BAL with a reduction in total cells and neutrophils in the CLP+BMS group compared to the CLP group (data not shown). Consistent with reduced neutrophilic lung inflammation, levels of the NF-*κ*B-dependent neutrophil chemotactic chemokine KC were lower in serum and BAL fluid in the CLP+BMS group compared to the CLP group (Figures 3(c) and 3(d)). Of other cytokines tested, MCP-1 was reduced, while IL-10 was increased in serum from mice treated with BMS-345541 following CLP compared to mice that underwent CLP followed by vehicle (Figures 3(e) and 3(f)). We also found a trend towards decreased IL-6, IL-17, and MIG in serum from mice treated with CLP+BMS compared to mice treated with CLP (+vehicle) (data not shown). In BAL fluid, levels of these cytokines were similar in both groups (CLP+BMS and CLP) at 24 hours after CLP (data not shown). 

**Figure 4:**
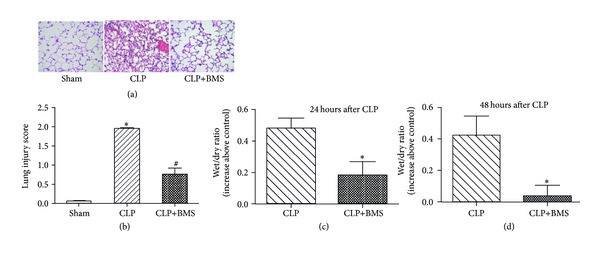
NF-*κ*B inhibition blocks lung injury and reduces apoptosis after CLP. (a) and (b) Histological evaluation of lung injury on H&E stained lung sections at 48 hours after CLP, *P < 0.05 for CLP versus sham laparotomy controls (Ctrl); ^#^ *P* < 0.05 for CLP+BMS versus CLP. (c) and (d) Lung edema as measured by wet/dry ratio at 24 and 48 hours after CLP, reported as increase above sham laparotomy controls. *N* = 5 per group; *P < 0.05 for CLP+BMS versus CLP. Results are presented as mean ± SE.

Since BMS-345541 inhibited NF-*κ*B activation and downstream proinflammatory cytokine responses, we asked whether BMS-345541 treatment would reduce CLP-induced lung injury. Mice treated with CLP developed characteristic histological changes of lung injury at 48 hours, including inflammatory cell infiltration, vascular congestion, alveolar edema, interalveolar septal thickening, and hemorrhage (Figures 4(a) and 4(b)). In contrast, mice treated with BMS-345541 following CPL showed preserved alveolar architecture with minimal edema and septal thickening. To quantify lung edema, we measured wet-to-dry ratios in lungs following CLP at 24 and 48 hours. The increased wet-to-dry ratio observed in CLP group was significantly attenuated in the CLP+BMS group, consistent with the histological changes observed in the lungs of mice from each treatment group (Figures 4(c) and 4(d)). Together, these studies indicate that pharmacologic NF-*κ*B inhibition initiated 2 hours after CLP reduces lung inflammation and prevents injury.

### 3.3. BMS-345541 Treatment following CLP Does Not Impair Bacterial Host Defense

**Figure 5:**
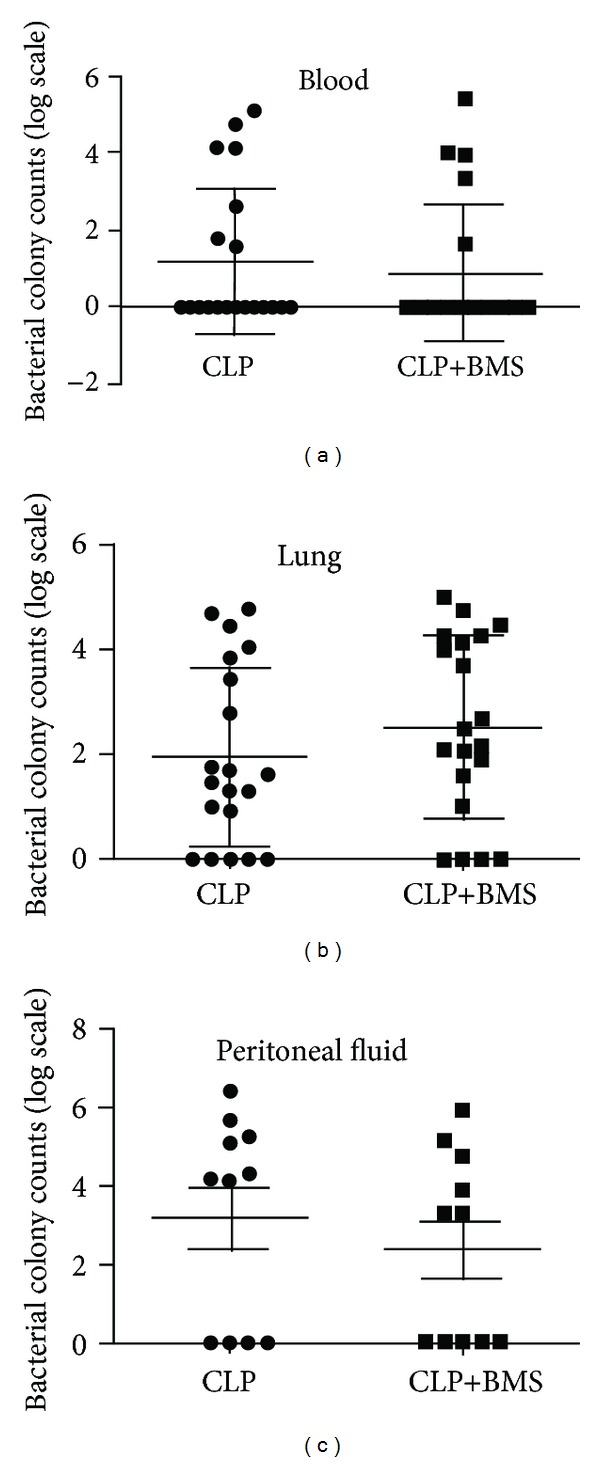
Inhibition of NF-*κ*B does not affect bacterial clearance after CLP. Bacterial colony counts in mice subjected to CLP+BMS and CLP+vehicle were assessed on samples from (a) blood (*N* = 20 per group), (b) lung tissue (*N* = 20 per group), and (c) peritoneal fluid (*N* = 11 per group) obtained at 24 hours after CLP. *X*-axis represents different groups and *y*-axis represents log scale for bacterial colony counts isolated from different sites. Results were represented as mean ± SE.

Although NF-*κ*B pathway activation plays an important role in host defense, we postulated that short-term NF-*κ*B inhibition initiated after the onset of sepsis-induced inflammation would limit downstream organ injury without impairing host defense against bacterial infection. To test this hypothesis, we performed bacterial colony counts from blood (*n* = 20 per group), lung tissue (*n* = 20 per group), and peritoneal fluid (*n* = 11 per group) at 24 hours in mice treated with CLP+BMS or CLP (+vehicle). Substantial intragroup variability in bacterial recovery from these sites occurred in both groups. We found no significant differences in colony counts from blood, lung tissue, or peritoneal fluid between BMS-345541- or vehicle-treated mice following CLP (Figures 5(a)–5(c)). These findings suggested that host defense was not impaired by BMS-345541 treatment.

**Figure 6:**
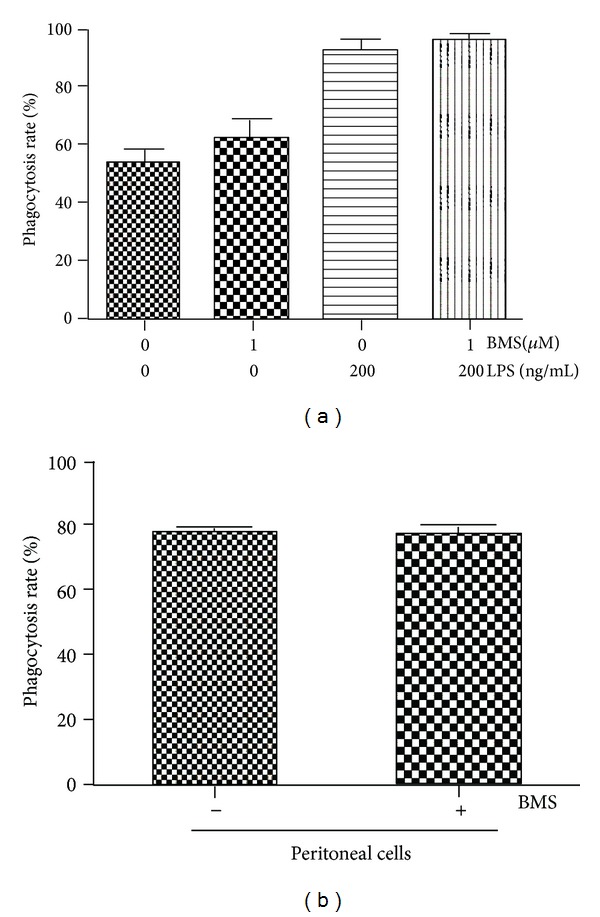
Treatment with BMS-345541 does not affect bacterial phagocytosis. (a) Phagocytosis of GFP-labeled *E. coli* by RAW264.7 macrophages was measured by counting fluorescent cells at 1 hour after adding bacteria. BMS-345541 and LPS were added to culture medium 1 hour prior to bacteria. (b) Cells were obtained by peritoneal lavage at 3 hours after CLP and bacterial phagocytosis was measured as described above. Results are presented as mean ± SE from 3 independent experiments.

Because macrophages play an important role in host defense against bacterial peritonitis we wondered whether NF-*κ*B inhibition affects bacterial uptake and killing by macrophages. We treated RAW264.7 macrophages with BMS-345541 to evaluate if NF-*κ*B inhibition affects macrophage phagocytosis. In preliminary studies, we found that BMS-345541 (1 *μ*M) inhibited LPS-induced NF-*κ*B activation without affecting cell survival. Pretreatment with LPS substantially increased phagocytosis of *E. coli*-GFP by RAW264.7 macrophages, but BMS-345541 treatment did not alter phagocytosis with or without LPS pretreatment (Figure 6(a)). Since neutrophils are also important for bacterial killing, we conducted an *ex vivo* experiment to evaluate the ability of innate immune cells in the peritoneum to phagocytose bacteria. Peritoneal cells (approximately 50% macrophages, 40% neutrophils at this time point after CLP) were harvested at 3 hours after CLP and incubated with *E. coli*-GFP+BSM-345541 or *E. coli*-GFP for 1 hour. As shown in Figure 6(b), BMS-345541 did not alter the ability of these cells to phagocytose bacteria. Together, these studies show that short-term inhibition of NF-*κ*B does not impair bacterial clearance following CLP and suggest that bacterial phagocytosis by innate immune cells is not impaired by NF-*κ*B inhibition.

### 3.4. NF-*κ*B Inhibition after CLP Does Not Affect Mortality

**Figure 7:**
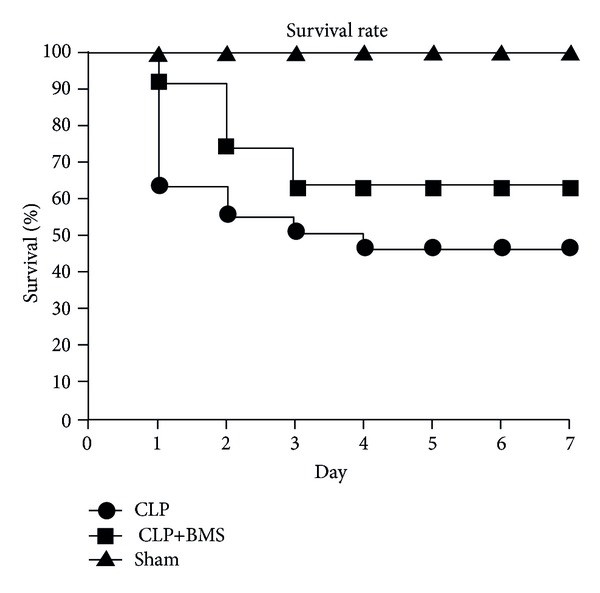
NF-*κ*B inhibition after CLP does not alter survival. Kaplan-Meier curves were plotted for mice subjected to CLP (+vehicle), CLP+BMS, or sham laparotomy (*n* = 25 per group). Log-rank test comparing CLP+vehicle and CLP+BMS: *P* = 0.144.

Although death in mouse sepsis models is thought to be predominantly due to shock rather than acute lung injury [21], we wanted to make sure that short-term treatment with BMS-345541 did not have a negative impact on survival after CLP. In his study, 25 mice per group were subjected to CLP (+vehicle), CLP+BMS, or sham laparotomy and followed for one week. At 7 days after CLP, all mice in the sham (control) group survived, compared to 12/25 in the CLP group and 18/25 in the CLP+BMS group (Figure 7). This study shows that systemic NF-*κ*B inhibition for a defined period of time after the onset of polymicrobial sepsis results in a trend towards improved survival, as opposed to a detrimental effect that would be expected if NF-*κ*B was required for bacterial clearance in this model.

## 4. Discussion

In these studies, we investigated the effects of inhibition of NF-*κ*B over a short, defined time period after the onset of sepsis. We found that a specific IKK inhibitor (BMS-345541) effectively suppressed NF-*κ*B activation and attenuated lung inflammation and injury. Importantly, bacterial clearance was not impaired by inhibiting NF-*κ*B during this time frame, and there was a trend towards increased survival after BMS-345541 treatment. Together, these findings suggest that inhibition of NF-*κ*B activation after the onset of sepsis might be an option for suppressing the systemic inflammatory response and downstream organ injury caused by microbial infection. 

Common models of sepsis include administration of exogenous toxins such as LPS, administration of viable bacteria, and polymicrobial infection resulting from CLP. In contrast to the rapid inflammatory response induced by systemic LPS treatment, CLP results in prolonged cytokine production resulting from peritoneal infection by enteric bacteria, which mimics abdominal sepsis in humans [22]. In previous studies, targeting NF-*κ*B during sepsis has resulted in variable outcomes that may be due in part to the different animal models used in these studies. For example, downregulation of NF-*κ*B activity can lead to improved survival in noninfectious endotoxemia models [6]. In combination with antibiotics, NF-*κ*B downregulation has been reported to increase survival following CLP [12]. However, NF-*κ*B inhibition in infection-related models can impair host defense, particularly in genetic models with constitutive defects in NF-*κ*B signaling, and reduce survival [14, 23]. 

In our studies, we used short-term targeted NF-*κ*B inhibition with the goal of reducing acute lung injury following CLP. Acute lung injury is a major clinical problem in the setting of human sepsis that results in respiratory failure and lacks specific treatments other than lung protective ventilator strategies. In rodent models of CLP, lung injury is relatively mild and is not thought to substantially contribute to mortality, which is primarily due to shock [21]. Therefore, it is not surprising that protection from acute lung injury following this dosing regimen of BMS-345541 was not associated with a reduction in mortality. Viewed in this context, our studies provide proof-of-concept that short-term NF-*κ*B inhibition (after onset of sepsis but prior to development of lung injury) could be beneficial in preventing acute lung injury without negative effects on bacterial clearance or mortality. Consistent with our findings, it has been suggested that partially inhibiting NF-*κ*B activation using nonspecific methods, such as elevated HSP70 expression and glutamine treatment, could result in reversal and/or prevention of lung injury in animals subjected to CLP [24–26].

In our studies, inhibition of NF-*κ*B activity appeared to lack detrimental effects on bacterial host defense. There are several potential explanations for this finding. First, treatment with BMS-345541 was begun at 2 hours after CLP and likely required several hours after the onset on infection to achieve substantial NF-*κ*B inhibition. Therefore, one would not expect the production of important proximal cytokines like TNF*α* and IL-1*β* to be reduced. The early wave of cytokines production, which occurs within the first 1-2 hours after infection, likely leads to early neutrophil recruitment and activation of mononuclear phagocytes that play critical roles in host defense. Second, only a partial and transient NF-*κ*B activation was achieved in these studies. When administered by the oral route, BMS-345541 has near complete absorption and bioavailability with a 2.2-hour half-life [27]. The relatively short half-life of BMS-345541 *in vivo* necessities repetitive dosing, as it was done in this study, and limits the ability to provide prolonged, high-level NF-*κ*B inhibition in tissues. Although this is a potential limitation of currently available compounds targeting the NF-*κ*B pathway, it may be beneficial in maintaining host defense functions. Third, we used a moderate CLP model for our studies. Differences in the severity of the CLP procedure can result in different degrees of peritonitis and different survival rates [17]. Although we did not test this possibility, NF-*κ*B inhibition might be detrimental in the setting of overwhelming peritoneal sepsis. Fourth, we found that NF-*κ*B inhibition with BMS-345541 did not affect basal or inducible phagocytosis of bacteria by macrophages and neutrophils. Together, a number of factors may account for the preservation of bacterial host defense in our studies despite the functional inhibition of NF-*κ*B. In these studies, we only tested one regimen of NF-*κ*B inhibition using a single agent. In the future, a more comprehensive assessment is warranted using a variety of sepsis models to better define the optimal timing and degree of NF-*κ*B inhibition to minimize lung injury while preserving host defense.

## 5. Conclusions

In conclusion, our study indicates that inhibition of NF-*κ*B for a defined time period after the onset of sepsis using a specific IKK inhibitor (BMS-345541) is beneficial in limiting lung injury without compromising host defense even in the absence of concurrent antibiotic treatment. We suggest that this approach could hold promise in humans with sepsis, where targeted therapies to limit the down-stream organ injury are needed.

## Abbreviations

ALI: Acute lung injury
LPS: Lipopolysaccharide
CLP: Cecal ligation and puncture
NF-*κ*B: Nuclear factor-*κ*B
ELISA: Enzyme linked immunosorbent assay
H&E: Hematoxylin and eosin
IL: Interleukin
MCP-1: Monocyte chemotactic protein-1
TNF: Tumor necrosis factor
PBS: Phosphate-buffered saline
IAS: Interalveolar septa.
